# Quality of life in intermittent exotropia for Korean children and their parents

**DOI:** 10.1186/s12886-023-02919-z

**Published:** 2023-04-26

**Authors:** Jin Seon Oh, Jae Ho Jung, Hyun Jin Shin

**Affiliations:** 1 School of Medicine, Konkuk University, Seoul, Republic of Korea; 2 Department of Ophthalmology, Seoul National University Hospital and Seoul National University College of Medicine, Seoul, Republic of Korea; 3 Department of Ophthalmology, Research Institute of Medical Science, Konkuk University Medical Center, Konkuk University School of Medicine, Seoul, Republic of Korea

**Keywords:** Deviation angle, Intermittent exotropia, Intermittent Exotropia Questionaire, Quality of life, Streoacuity

## Abstract

**Background:**

Patients with strabismus are more likely to have mental health problems, including high rates of depressive symptoms and social phobia. Intermittent exotropia (IXT) typically occurs in early childhood and is more common in Asian populations. We aim to assess the health-related quality of life (HRQOL) concerns in children with intermittent exotropia (IXT) using the Intermittent Exotropia Questionaire (IXTQ), and their associations with the clinical severity of IXT and the parents’ HRQOL concerns.

**Methods:**

IXT, defined as both distance and near exodeviation ≥ 10 prism diopters were eligible for inclusion. The final IXTQ score is calculated using the mean score for all items, and ranges from 0 (worst HRQOL) to 100 (best HRQOL). The correlations of child IXTQ scores with their deviation angle and stereoacuity were measured, as were those with their parent’s IXTQ scores.

**Results:**

One hundred twenty-two children with IXT (aged 5–17 years) and one parent for each child completed the child and parent IXTQ, respectively. The greatest HRQOL concern for each child with IXT and their parent was “Worry about eyes” (frequency 88%, score 35.0 ± 27.8). Lower child IXTQ scores were associated with a larger distance (*r* = 0.24, *p* = 0.007) and near deviation angle (*r* = 0.2, *p* = 0.026). “It bothers me because I have to wait for my eyes to clear up” and “Waiting for their eyes to clear up” were more common in children with a larger deviation angle (both *p < 0.05*). The parent IXTQ scores (52.1 ± 25.3) were lower than the child ones (79.7 ± 15.8) and showed a positive correlation with child IXTQ scores (*r* = 0.26, *p* = 0.004). Lower parent IXTQ scores were associated with poor distance stereoacuity (*r* = 0.23, *p* = 0.01).

**Conclusion:**

The HRQOL of IXT children was positively related to that of their parents. A larger deviation angle and worse distance stereoacuity function may predict more-negative impacts on children and their parents, respectively.

**Supplementary Information:**

The online version contains supplementary material available at 10.1186/s12886-023-02919-z.

## Background

Intermittent exotropia (IXT) is characterized by intermittent outward deviation of the eyes and is the most common form of strabismus that affects 1–2% of the population [1]. The onset of IXT typically occurs in early childhood and is more common in Asian populations, and constitutes half of the cases of primary horizontal strabismus [2]. Increases in the angle and frequency of deviation can worsen binocular vision and become a cosmetic problem. Additionally, patients with strabismus are more likely to have mental health problems [3], including high rates of depressive symptoms and social phobia [4].

Considering its negative psychosocial impact on children [5], not only the clinical severity but also the health-related quality of life (HRQOL) of the patients should be assessed in the management of IXT. Previous studies found that HRQOL was worse in both children with IXT and their parents [6], and that a higher severity of IXT was associated with a worse HRQOL of patient and their parent [7].

The Intermittent Exotropia Questionnaire (IXTQ) is a condition-specific, patient-derived validated HRQOL questionnaire for children with IXT and their parents [8]. Although other questionnaires such as PedsQL can be used to assess HRQOL in children with strabismus, they are less sensitive and specific to IXT [6, 9]. IXTQ is a reliable and valid scale that captures the influence of IXT on multiple aspects of the quality of life, including social function.

Parental HRQOL is not always positively related to Patient HRQOL. Previous study reporting the health-related quality of life of children with congenital cataract found that there was considerable variation in agreement of scores reported by individual child–parent pairs. The aims of this study were to identify the specific HRQOL concerns in children with IXT and their parents using the IXTQ, and the relationship between HRQOL and the clinical severity of IXT. In addition, we determined the relationship between the HRQOL of IXT children and that of their parents. The results of this study will help clinicians and parents to consider physical and psychosocial aspects together when managing children with IXT.

## Methods

This study prospectively recruited 122 children with IXT aged 5–17 years from Konkuk University Hospital Seoul, South Korea. between January 2017 and January 2020. The study was performed in accordance with the principles of the Declaration of Helsinki, and its protocol was approved by the institutional review board and ethics committee at Konkuk University Medical Center (approval no. 2020-07-015). For each child, one accompanying parent or legal guardian was also recruited. All children and their parents provided written informed consents to participate in this study.

Included subjects were consecutive children with intermittent XT in our clinical practice and were referred for a variety of reasons by their primary care giver (pediatrician or family practitioner) or for parental or child concerns. The following inclusion criteria were applied: (1) both distance and near exodeviations measured by prism and alternate cover test > 10 prism diopters (PD) and distant control ≥ 1, (2) best corrected visual acuity in the worse eye of 20/25 (logMAR of 0.1) or better, and (3) visual acuity difference between eyes not greater than two lines. The following exclusion criteria were applied: (1) other ocular problems such as nystagmus, limitation of eye movements, or amblyopia, (2) eyelid problems such as epiblepharon or ptosis, (3) previous surgery or botulinum injection for strabismus, (4) birth weight < 1500 g, (5) birth at a gestational age of < 34 weeks, (6) developmental delay, systemic illness, syndrome, or learning disability, (8) history of any psychiatric illness or medication, (9) IXT onset before 2 years of age, (10) abnormal head posture such as head tilt, face turn, chin elevation and chin depression, or (11) history of eye convergence exercises or patching that could impact IXTQ score.

### Assessment of HRQOL

All participants and their parents were asked to complete the IXTQ. The child and parent IXTQ versions consist of 12 and 17 questionnaire items, respectively. Each question is scored using the following five-point Likert-type scale: never (score 100), almost never (75), sometimes (50), often (25), and almost always (0). When applying the child IXTQ to children aged 5–7 years, only three levels are used: not at all (100), sometimes (50), and a lot (0). The final IXTQ score is calculated as the mean score for all items, and ranges from 0 (worst HRQOL) to 100 (best HRQOL) [10]. Children and parents completed the respective child and parent versions of the IXTQ without any communication. If the child or their parents had any problems in understanding the questionnaire, a verbal interview was conducted without any explanation or elaboration [8]. The English version of IXTQ was translated into Korean. Intermittent Exotropia Questionnaire (IXTQ) was independently translated by two ophthalmologists (J.H.J. and H.J.S). After merging the two translation, the first translation version was revised by an ophthalmologist who is fluent in both languages. If the word concept did not match, it was reviewed again and corrected. The English questionnaire and the first translation version were asked to be completed on different days by subjects who were fluent in English interpretation. The final translation was completed by adjusting the vocabulary for the items showing significant differences. The mean score for each questionnaire item was calculated for the child and parent questionnaires. In addition, the proportion of high-frequency responses (defined for this study as “sometimes,” “often,” or “a lot” / “almost always”) was calculated for each item, and they were then ranked accordingly.

### Assessment of the severity of IXT

The severity of IXT in our patients was assessed by the angle of the deviation and stereoacuity. Ocular alignment was assessed using the cover/uncover test and the alternate prism and cover test. The deviation angle was measured using the prism alternate cover test at distance (4 m) and near (33 cm). The deviation angle was measured 1 h after applying monocular occlusion. The near and far stereoacuities were measured using the Titmus fly test and Randot stereoacuity test, respectively. Stereoacuity values were transformed into log-arcsec values for the purpose of the analysis; if the patient had no measurable stereoacuity, a stereoacuity of 3.2 log arcsec was assigned [8].

### Statistical analysis

All calculations and statistical analyses were performed using SPSS for Windows (version 26.0, SPSS, Chicago, IL, USA). The Shapiro-Wilk test was used to determine whether the data conformed to a parametric (Gaussian) or nonparametric (non-Gaussian) distribution. Child and parent scores for individual items were compared using Wilcoxon rank-sum tests, and the proportions of high-frequency responses were compared between children and parents for each item using Fisher’s exact tests. The mean scores for the child and parent HRQOL were compared using the two-tailed independent-samples *t*-test. The relationship between child and parent IXTQ scores was assessed with a multiple linear regression model. The correlations of IXTQ scores with the exodeviation and stereoacuity were evaluated using Pearson’s correlation. The mean ± standard deviation (SD) and the median (interquartile range) are presented. A P value < 0.05 was considered statistically significant.

## Results

**Fig. 1:**
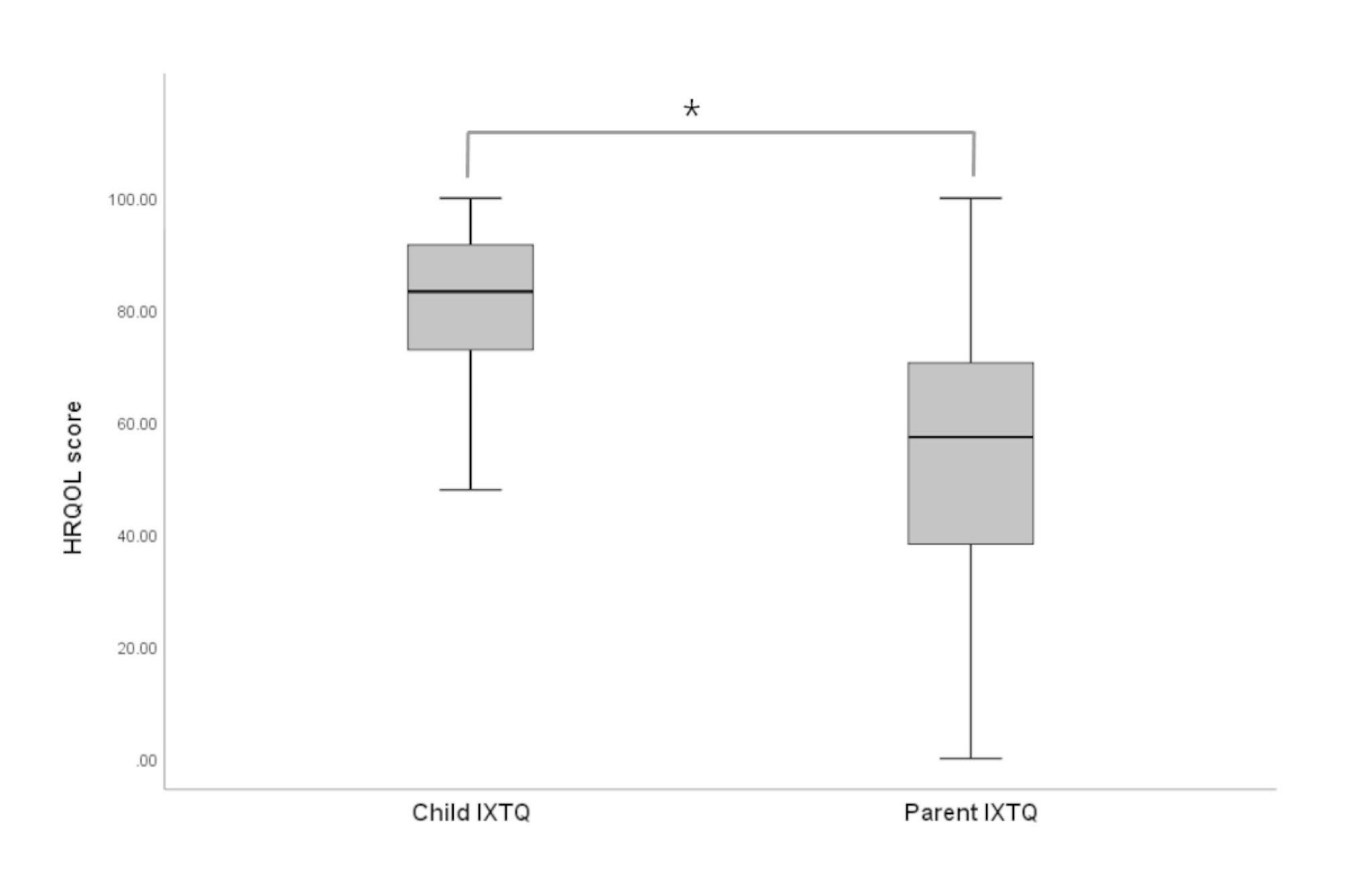
Comparison between scores for children and parents on the Intermittent Exotropia Questionnaire (IXTQ). The possible score ranges from 100 for the best health-related quality of life (HRQOL) to 0 for the worst HRQOL. Each box plot shows the median, first and third quartiles, and range

This study included 122 children with IXT aged 8.6 ± 2.4 years (range of 5–17 years) and their accompanying parents. The demographics, measured strabismus values of the enrolled children are listed in Table 1. The IXTQ score was 79.7 ± 15.8 (median [IQR], 81.3 [70.8–91.7]) for the child scale and 52.1 ± 25.3 (median [IQR], 57.4 [38.2–70.6]) for the parent scale (Fig. 1). There were no significant correlations between children’s age and their deviation angle or near and far stereoacuities. Although, mean Child IXTQ scores and frequencies were mostly similar for younger (5 - <8 years) and older (8 - <17 years) children on most items, significant differences were observed between younger and older children for “I worry about my eyes” and “It bother bothers that I have to shut one eye when it is sunny” (Supplementary Table 2). We also found no noticeable difference between the concerns reported by females and males.

**Table 1:** Characteristics of 122 intermittent exotropia children Data are *n* (%) or range (mean ± SD) values

Characteristic	Value
***Demographics***	
Age, years	
5–7	35 (28.6%)
8–17	87 (71.4%)
Gender	
Male	67 (54.9%)
Female	55 (45.1%)
Accompanying parent	
Father	27 (22.1%)
Mother	93 (76.2%)
Guardian	2 (1.7%)
***Strabismus measurements***	
Exodeviation	
Distance	10–50 (21.2 ± 7.9)
Near	10–50 (28.5 ± 8.2)
Distance stereoacuity, arcsec	
≤80	45 (34.8%)
100–200	31 (26.8%)
400	16 (12.5%)
> 400	30 (25.9%)
Near stereoacuity, arcsec	
≤80	66 (54.1%)
100–200	30 (24.6%)
400	22 (18.0%)
>400	4 (3.3%)

### IXTQ items: mean scores and proportions of high-frequency responses

For the child IXTQ, the item with the greatest HRQOL impact was “I worry about my eyes” (frequency 57%, score 62.9 ± 31.2) followed by “It bothers me that I have to shut one eye when it is sunny” (43%, 67.8 ± 34.4) and “I am bothered when my parents say things about my eyes” (42%, 69.7 ± 33.1). The items with the least HRQOL impact were “My eyes make it hard for me to make friends” (3%, 96.9 ± 12.7) and “Kids tease me because of my eyes” (8%, 93.9 ± 18.3). Supplementary Table 1 lists all of the responses to the questions on the child HRQOL questionnaire.

For the parent IXTQ, the item with the greatest HRQOL impact was “I worry about my child’s eyes” (frequency 88%, score 35.0 ± 27.8), followed by “I worry about my child’s eyesight long term” (81%, 37.3 ± 30.5). The item with the least HRQOL impact was “It worries me what others will think about my child because of his/her eyes” (44%, 61.9 ± 33.0), followed by “I worry about my child’s ability to make friends” (41%, 63.9 ± 33.4), Supplementary Table 3 lists all of the responses to the questions on the parent HRQOL questionnaire.

### Association between child and parent HRQOL

**Fig. 2:**
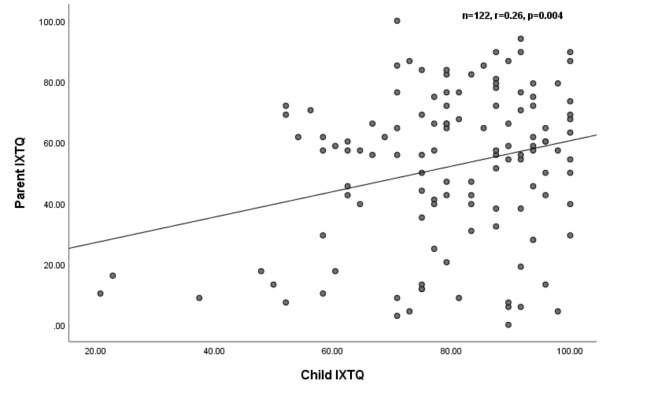
Association between child and parent IXTQ scores

Figure 2 shows the correlation between the child and parent IXTQ scores. As the HRQOL of children with IXT deteriorated, that of the parents also worsened (*r* = 0.26, *p* = 0.004). The independent variable was “I worry that my child will be less independent because of his/her eyes” for child IXTQ and “I am bothered when my parents say things about my eyes”. We conducted multivariate linear regression analysis to evaluate the associations between (1) child IXTQ items and parent IXTQ scores, and (2) parent IXTQ items and child IXTQ scores. Among the child IXTQ items, “I am bothered when my parents say things about my eyes” was significantly associated with parent IXTQ scores (*p* = 0.003). Among the parent IXTQ items, “I worry that my child will be less independent because of his/her eyes” was significantly associated with child IXTQ scores (*p* < 0.0001).

### Correlation between HRQOL and severity of strabismus

**Fig. 3:**
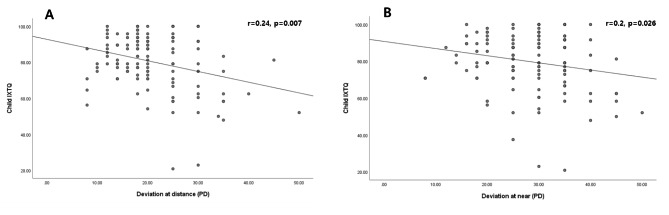
Correlations of child IXTQ scores with distance (A) and near (B) exodeviations

There were positive correlations of the child IXTQ scores with both the distance and near exodeviations (Fig. 3), while parent IXTQ scores were not significantly correlated with the exodeviation of the children. Larger distance (*r* = 0.24, *p* = 0.007) and near (*r* = 0.2, *p* = 0.026) exodeviations were associated with a worse child IXTQ score. The mean value of the deviation angle was used to divide the patients into two groups for each type of exodeviation: severe (≥ 25 PD) and mild (< 25 PD) distance exodeviations, and severe (≥ 35 PD) and mild (< 35 PD) near exodeviations. To identify which child IXTQ items were associated with the severity of deviation, we compared the scores for each item between the two groups. The mean scores for “It bothers me because I have to wait for my eyes to clear up” (*p* = 0.002 and *p* = 0.02 for distance and near exodeviations, respectively) and “I am bothered when grownups say things about my eyes” (*p* = 0.004 and *p* = 0.03) were significantly lower in the severe-deviation group than in the mild-deviation group (Supplementary Tables 4 and 5).

**Fig. 4:**
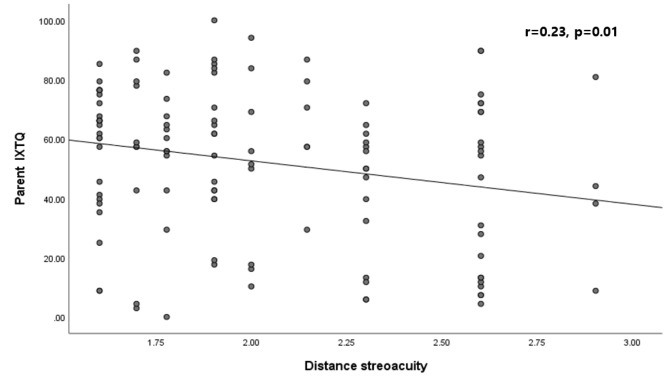
Correlations of parent IXTQ scores with distance stereoacuity in children with intermittent exotropia

With regard to stereopsis, there was no significant correlation between child IXTQ scores and stereoacuity. However, there was a positive correlation between the parent IXTQ scores and distance stereoacuity (*r* = 0.23, *p* = 0.01) (Fig. 4), while near stereoacuity was not significantly correlated with parent IXTQ scores (*r* = 0.02, *p* = 0.13). A worse distance stereoacuity in children with IXT was associated with a worse parent IXTQ score. We divided also the patients into two groups based on the distance stereoacuity: good (≤ 100 arcsec) and poor (> 100 arcsec). To evaluate the parent IXTQ items associated with distance stereoacuity, we compared the mean scores and frequencies for each item between the groups with good and poor distance stereoacuity. The items of “I worry that my child will have permanent damage to his/her eyes” and “I worry about how my child’s eyes will affect him/her socially” had the most-significant *p* values between the groups (Supplementary Table 6).

## Discussion

We have identified a range of specific HRQOL concerns affecting children with IXT and their parents. The items with the greatest HRQOL impact were “I worry about my eyes (my child’s eye)” and “Shutting one eye when it is sunny.” Items related to making friends was of least concern. Also, there was a positive correlation between the child and parent HROQL scores (*r*^2^ = 0.07, *p* = 0.004). The deviation angle was associated with the child HRQOL score and the distance stereoacuity was associated with the parent HRQOL score.

Worrying about the eyes was also the greatest concern reported by both children and their parents. Parents experienced stress regarding the children’s problems, indicating that pediatric ophthalmologists should offer appropriate education to reduce the unnecessary worries of both the parents and their affected children, since loss of vision and depth perception rarely occur in patients with IXT. Although surgery is the mainstay of treatment for IXT and IXT can recur after surgery, strabismus surgery is generally a safe procedure. Detailed explanations about treatment and prognosis would be very helpful for improving the quality of life of affected children and their parents. In addition, older children had worse HRQOL than younger children for the item, “I worry about my eyes” because we predicted that older children would be more self-aware.

Closure of one eye in bright sunlight was the second most common complaint contributing to the impairment of children’s HRQOL. Sensitivity to sunlight is a well-recognized specific feature of childhood IXT [10, 11]. Monocular eye closure or sensitivity to sunlight is a common complaint in children with IXT. It has been explained that bright sunlight overloads the retina so as to somehow disrupt fusion, causing the deviation to become manifest. Patients with IXT whose eyes might be disassociated close one eye to avoid diplopia and visual confusion [12]. Thus, clinicians should understand that monocular closure is a common phenomenon in children with IXT and that it makes them feel uncomfortable. The use of sunglasses or tinted prescription lenses during outdoor activities may help to improve their photosensitivity by decreasing the intensity of light reaching the retina. In addition, younger children tend to show high-frequency responses for the item “It bothers me that I have to shut one eye when it is sunny” (Supplementary Table 2). It is possible that older children have learned to control their deviation better in sunny weather, and therefore the sunny condition does not bother them as much.

It was particularly interesting that teasing and difficulty making friends were of least concern among both children and their parents. This finding is consistent with previous studies in Japan that have shown that children with IXT experience a surprising lack of teasing and bullying [10]. On the other hand, some previous studies have found that negative social bias and teasing are experienced by children with noticeable strabismus [13, 14]. It’s also likely that peers are less aware of exodeviation in children with IXT because they can typically regulate it at conversational distances. However, if misalignment associated with the exotropia becomes manifest from the latent or intermittent form, clinicians should consider its negative social impact and surgical intervention.

The HRQOL of children was correlated with that of their parents (*r*^2^ = 0.07, *p* = 0.004). In the multivariate linear regression analysis, items of “I am bothered when my parents say things about my eyes” and “I worry that my child will be less independent because of his/her eyes” were significantly associated with poor parent and child IXTQ scores, respectively. Therefore, it would be advisable for parents to not point out their children’s eye problems. In addition, ophthalmologists should advise parents that IXT is unlikely to affect their children’s vision and consequently their independence.

We found that the child HRQOL scores were associated with the deviation angle (at distance, *r*^2^ = 0.06, *p* = 0.007 vs. at near, *r*^2^ = 0.04, *p* = 0.026). Our results are consistent with Wang et al. recently finding that child IXTQ scores were correlated with both distance and near exodeviations [7]. Also, we found that the main concerns reported by children with severe deviation angles were them waiting for their eyes to improve and adults commenting about their eyes. These findings support previous reports of individuals with larger deviation and worse control in exotropia experiencing greater disturbances in visual performance [7]. A larger exodeviation makes the accommodative convergence required to maintain ocular alignment and binocular vision more difficult to achieve [15]. This explains why children with a large deviation angle were concerned about waiting for their eyes to improve. Regarding adults commenting about their eyes, it is unsurprising as several previous studies also found that those children with noticeable exotropia were subjected to negative social bias from their peers and teachers [13, 14].

Another interesting finding was that parent HRQOL scores were associated with distance stereoacuity (*r*^2^ = 0.05, *p* = 0.01) but not near stereoacuity (*p* = 0.13). This was supported by previous findings of poor distance stereoacuity appearing to be a better objective parameter than near stereoacuity for poor control of the exotropic deviation [16, 17]. Patients with IXT could maintain stable near stereoacuity, with its deterioration being infrequent [18]. This could be interpreted as indicating that children with poor distance stereoacuity have poor control of exotropic deviation and that their parents would detect the deviation more frequently and therefore worry more. This hypothesis was supported by a previous report of decreased distance stereoacuity probably representing poor control of the deviation, which is often considered when deciding who should undergo surgical correction [17]. Thus, distance stereoacuity provides a useful means of assessing the parent’s HRQOL as well as their children’s sensory visual function in IXT.

This study found that child HRQOL scores were associated with exodeviation but not stereoacuity, whereas parent HRQOL scores were associated with distance stereoacuity but not exodeviation. Wang et al. found that the child HRQOL and parent HRQOL were correlated with both the deviation angle and stereoacuity [7]. Although no completely consistent correlation was found between HRQOL, stereoacuity, and deviation angle, it appears that objective clinical measurements reflect the subjective HRQOL of children and their parents [19].

This study had several limitations. The setting was a tertiary-care institution which may have resulted in selection bias regarding the severity of strabismus. Quality of life is primarily attributed to socioeconomic factors, and previous studies have shown that a lower socioeconomic status is associated with a worse HRQOL [20]. However, the parental education level and economic status were not analyzed in the current study. In most cases the child’s parent included in this study was the mother, and so evaluating differences between the concerns reported by fathers and mothers may therefore be worthy of further study. We evaluated angle of deviation after 1 h of monocular occlusion for measuring maximum angle of deviation and differentiating between the true divergence excess and pseudo-divergence excess type. However, this conditions, eliminated fusion with occlusion, are not truly the ones which the patients experiment throughout their daily life. This study included only Korean subjects, and IXTQ would vary between different cultures, meaning that the correlation between child and parent HRQOL scores could differ in Western countries. Also, spectacles wear can impact IXTQ score. Thus, future studies should examine cultural differences and the effect of spectacles on the HRQOL of children and their parents.

In conclusion, the greatest HRQOL concerns for children with IXT were worrying about their eyes and shutting one eye when sunny. The child HRQOL score is associated with the parent HRQOL score. A larger deviation angle and worse distance stereoacuity function may predict more-negative impacts on the lives of children and their parents, respectively. Evaluation of HRQOL concerns using the IXTQ may be helpful in the clinical care of individual patients and their parents.

## Electronic supplementary material

Below is the link to the electronic supplementary material.

Supplementary Material 1

## Data Availability

The datasets used during the current study are available from the corresponding author on reasonable request.
