# Supplementary material for: Quality of life in intermittent exotropia for Korean children and their parents
Source: BMC Ophthalmol. 2023 Apr 26;23:185. doi: 10.1186/s12886-023-02919-z (PMC10134591; doi:10.1186/s12886-023-02919-z)
Supplement: Supplementary file 1 — Supplementary Material 1 [file 12886_2023_2919_MOESM1_ESM.docx]

**Supplementary tables**

**Supplementary Table 1.** Scores for individual items of the child Intermittent Exotropia Questionnaire (IXTQ).

|  | Child IXTQ score | |
| --- | --- | --- |
| IXTQ item | Score | frequency |
| 1. I worry about my eyes | **62.9 ± 31.2** | **0.57** |
| 2. It bothers me that people wonder what is wrong with my eyes | 73.8 ± 31.5 | 0.34 |
| 3. It bothers me because I have to wait for my eyes to clear up | 74.4 ± 30.4 | 0.34 |
| 4. Kids tease me because of my eyes | **93.9 ± 18.3** | **0.08** |
| 5. I am bothered when grownups say things about my eyes | 74.0 ± 30.2 | 0.37 |
| 6. I am bothered when my parents say things about my eyes | **69.7 ± 33.1** | **0.42** |
| 7. It bothers me that I have to shut one eye when it is sunny | **67.8 ± 34.4** | **0.43** |
| 8. I feel different from other kids because my eyes go in and out | 88.1 ± 21.4 | 0.15 |
| 9. I worry about what other people think of me because of my eyes | 81.1 ± 26.6 | 0.25 |
| 10. My eyes make it hard to look people in the eye | 89.5 ± 23.1 | 0.11 |
| 11. It is hard to concentrate because of my eyes | 84.8 ± 29.0 | 0.16 |
| 12. My eyes make it hard for me to make friends | **96.9 ± 12.7** | **0.03** |

Data are mean±SD values and proportions of high-frequency responses

**Supplementary Table 2.** The mean scores and frequency on individual child intermittent cxotropia questionnaire (IXTQ) in sub-groups of younger versus older children

|  | Child IXTQ | | | |
| --- | --- | --- | --- | --- |
| IXTQ item | 5-<8  years  (n=35) | 8-<17  years  (n=77) | P value  (mean) | P value  (frequency) |
| 1. I worry about my eyes | 71.4±34.9(0.46) | **59.4±27.5(0.62)** | **0.03** | 0.1 |
| 2. It bothers me that people wonder what is wrong with my eyes | 77.1±32.8(0.37) | 75.0±28.7(0.3) | 0.4 | 0.4 |
| 3. It bothers me because I have to wait for my eyes to clear up | 77.1±30.5(0.4) | 72.1±30.3(0.33) | 0.3 | 0.4 |
| 4. Kids tease me because of my eyes | 98.6±8.5(0.03) | 93.8±19.1(0.08) | 0.1 | 0.4 |
| 5. I am bothered when grownups say things about my eyes | 75.7±32.9(0.4) | 72.4±29.7(0.35) | 0.3 | 0.6 |
| 6. I am bothered when my parents say things about my eyes | 67.1±36.3(0.51) | 72.1±29.8(0.36) | 0.7 | 0.1 |
| 7. It bothers me that I have to shut one eye when it is sunny | **62.8±39.0(0.54)** | 72.7±30.7(0.34) | 0.3 | **0.04** |
| 8. I feel different from other kids because my eyes go in and out | 90.0±23.6(0.17) | 88.3±18.4(0.12) | 0.2 | 0.5 |
| 9. I worry about what other people think of me because of my eyes | 82.8±29.6(0.29) | 82.8±22.7(0.2) | 0.4 | 0.3 |
| 10. My eyes make it hard to look people in the eye | 88.6±27.3(0.17) | 91.2±20.6**(0.07)** | 0.8 | 0.1 |
| 11. It is hard to concentrate because of my eyes | 85.7±33.4(0.17) | 85.1±27.3(0.14) | 0.2 | 0.7 |
| 12. My eyes make it hard for me to make friends | 95.7±18.7(0.06) | 97.4±9.6(0.03) | 0.7 | 0.6 |

**Supplementary Table 3.** Scores for individual items of the parent IXTQ.

|  | Parent IXTQ score | |
| --- | --- | --- |
| IXTQ item | Score | frequency |
| **1. I worry about my child’s eyes** | **35.0 ± 27.8** | **0.88** |
| 2. I worry that my child will be less independent because of his/her eyes. | 58.2 ± 35.7 | 0.52 |
| 3. I worry that my child will have permanent damage to his/her eyes. | 43.2 ± 33.7 | 0.69 |
| 4. I worry that my child does not see well. | 54.9 ± 35.3 | 0.53 |
| 5. I worry about how my child’s eyes will affect him/her socially | 55.7 ± 33.8 | 0.52 |
| 6. I worry that my child will get hurt physically because of his/her eyes. | 56.8 ± 34.3 | 0.51 |
| 7. I worry about the possibility of surgery. | 48.8 ± 31.5 | 0.70 |
| 8. I worry about my child becoming self-conscious because of his/her eyes. | 53.9 ± 33.6 | 0.56 |
| 9. I worry that my child will not be able to see the board at school. | 50.6 ± 33.0 | 0.66 |
| 10. I worry about other kids teasing my child because of his/her eyes. | 58.4 ± 34.0 | 0.50 |
| **11. It worries me what others will think about my child because of his/her eyes.** | **61.9 ± 33.0** | **0.44** |
| 12. I worry that my child’s eye condition will affect his/her personality. | 56.6 ± 34.4 | 0.53 |
| 13. I worry that my child’s eyes will affect his/her social life if nothing is done. | 47.7 ± 31.3 | 0.66 |
| **14. I worry about my child’s eyesight long term.** | **37.3 ± 30.5** | **0.81** |
| 15. I worry about my child’s depth perception. | 51.0 ± 33.3 | 0.59 |
| 16. I worry about whether my child should have surgery | 51.4 ± 31.9 | 0.66 |
| **17. I worry about my child’s ability to make friends.** | **63.9 ± 33.4** | **0.41** |

**Supplementary Table 4.** Comparison of child health-related quality of life (HRQOL) scores between severe (≥25 prism diopters [PD]) and mild (<25 PD) distance exodeviations. Only items with a significant *p* value are listed.

|  | Distance | | | |
| --- | --- | --- | --- | --- |
| Child IXTQ item | Severe  ≥25 PD  (n=46) | Mild  <25 PD  (n=76) | P value  (Mean) | P value  (Frequency) |
| 3. It bothers me because I have to wait for my eyes to clear up | 63.0±34.1(0.5) | 81.3±25.9(0.24) | 0.002 | 0.003 |
| 4. Kids tease me because of my eyes | 89.1±24.5(0.15) | 96.7±12.5(0.04) | 0.048 | 0.04 |
| 5. I am bothered when grownups say things about my eyes | 63.6±32.8(0.54) | 80.3±26.9(0.26) | 0.004 | 0.002 |
| 6. I am bothered when my parents say things about my eyes | 61.4±34.8(0.56) | 74.7±34.2(0.33) | 0.03 | 0.01 |
| 9. I worry about what other people think of me because of my eyes | 75.0±28.4(0.4) | 84.9±24.9(0.16) | 0.045 | 0.004 |

**Supplementary Table 5.** Comparison of child HRQOL scores between severe (≥35 PD) and mild (<35 PD) near exodeviations. Only items with a significant *p* value are listed.

|  | Near | | | |
| --- | --- | --- | --- | --- |
| Child IXTQ item | Severe  ≥35 PD  (n=40) | Mild  <35 PD  (n=82) | P value  (Mean) | P value  (Frequency) |
| 3. It bothers me because I have to wait for my eyes to clear up | 66.2±31.3(0.42) | 78.4±29.3(0.29) | 0.02 | 0.15 |
| 5. I am bothered when grownups say things about my eyes | 66.3±30.2(0.52) | 77.7±29.7(0.29) | 0.03 | 0.01 |

**Supplementary Table 6.** Comparison of parent HRQOL scores between children with good (≤100 arcsec) and poor (>100 arcsec) distance stereoacuity. Only items with a significant *p* value are listed.

|  | Distance | | | |
| --- | --- | --- | --- | --- |
| Parent IXTQ item | Good  ≤100 PD  (n=64) | Poor  >100 arc  (n=58) | P value  (Mean) | P value  (Frequency) |
| **3. I worry that my child will have permanent damage to his/her eyes** | **51.2±33.5(0.56)** | **34.5±32.0(0.83)** | **0.005** | **0.002** |
| 4. I worry that my child does not see well. | 61.3±33.0(0.48) | 47.8±36.7(0.59) | 0.04 | 0.3 |
| **5. I worry about how my child’s eyes will affect him/her socially** | **57.8±33.0(0.48)** | **43.5±32.3(0.7)** | **0.02** | **0.01** |
| 9. I worry that my child will not be able to see the board at school | 58.2±30.6(0.59) | 42.2±33.8(0.72) | 0.01 | 0.1 |
| 13. I worry that my child’s eyes will affect his/her social life if nothing is done. | 53.9±29.6(0.59) | 40.9±32.0(0.72) | 0.02 | 0.1 |
| 14. I worry about my child’s eyesight long term. | 43.0±30.4(0.76) | 31.0±29.7(0.86) | 0.03 | 0.2 |
| 15. I worry about my child’s depth perception. | 57.8±33.0(0.48) | 43.5±32.3(0.7) | 0.02 | 0.01 |
| 17. I worry about my child’s ability to make friends. | 57.3±35.0(0.53) | 57.3±35.0(0.53) | 0.04 | 0.008 |
